# Early Motor Signs in Autism Spectrum Disorder

**DOI:** 10.3390/children9020294

**Published:** 2022-02-21

**Authors:** Annio Posar, Paola Visconti

**Affiliations:** 1 IRCCS Istituto delle Scienze Neurologiche di Bologna, UOSI Disturbi dello Spettro Autistico, 40139 Bologna, Italy; paola.visconti@isnb.it; 2 Dipartimento di Scienze Biomediche e Neuromotorie, Università di Bologna, 40126 Bologna, Italy

**Keywords:** autism spectrum disorder, early diagnosis, motor signs

## Abstract

A growing number of literature data suggest the presence of early impairments in the motor development of children with autism spectrum disorder, which could be often recognized even before the appearance of the classical social communication deficits of autism. In this narrative review, we aimed at performing an update about the available data on the early motor function in children with autism spectrum disorder. Early motor impairment in these children can manifest itself both as a mere delay of motor development and as the presence of atypicalities of motor function, such as a higher rate and a larger inventory, of stereotyped movements both with and without objects. In the perspective of a timely diagnosis, the presence of early motor signs can be an important clue, especially in an individual considered at high risk for autism. Motor and communication (both verbal and non-verbal) skills are connected and a pathogenetic role of early motor dysfunctions in the development of autism can be hypothesized. From this, derives the importance of an early enabling intervention aimed at improving motor skills, which could also have favorable effects on other aspects of development.

## 1. Introduction

Autism Spectrum Disorder (ASD) is a clinical condition characterized by social communication and interaction deficits, as well as by restricted interests and repetitive behaviors, according to the criteria of the Diagnostic and Statistical Manual of Mental Disorders, Fifth Edition (DSM-5) [1]. ASD diagnosis is still clinical; apart from the core signs of autism, for some time a large number of heterogeneous signs of motor development impairment have been reported in infants and children with ASD. Below, we report some of the most relevant of these signs described in the literature during the past few years: delayed motor development; persistent asymmetry when lying on the stomach at 4 months of age; righting from the supine to the prone position moving all the body en bloc not in a corkscrew fashion; abnormal patterns of crawling; walking asymmetry; sequencing instead of superimposition of one movement on the other for example during gait; unusual positions of arms; poor coordination; muscle tone and reflex abnormalities; choreiform movement of extremities; impaired finger-thumb opposition; stereotyped movements of the body, limbs, and fingers, including hand flapping; unusual gait patterns, including walking on tiptoes; poor motor imitation; impairment of postural control [1,2,3,4,5,6]. An approximate idea of the prevalence of motor impairment in ASD is given by Ming et al., who in 154 children found: hypotonia in 51% of cases, apraxia in 34%, walking on tiptoes in 19%, and gross motor delay in 9% [7]. These percentages, however, are probably underestimated, due to the relatively high median age (6 years) of the series studied, while over time the early motor signs tend to reduce [8] and however, they often go unnoticed compared to what are considered the core signs of autism. Yet, despite all these data, to this day, motor function impairments are not considered as diagnostic criteria of ASD, so much so that they are included only in the associated clinical features of ASD, according to DSM-5 [1]. Particularly during the last years, a growing number of data have suggested the presence of mostly mild early motor impairments and/or atypicalities in the development of children with ASD that could be often recognizable even before the appearance of the classical ASD social communication deficits. Of course, this finding could be very important also in the perspective of an early diagnosis and a prompt enabling intervention.

In this narrative review, we aimed at performing an update about the available data on the early motor function in children with ASD.

## 2. Materials and Methods

**Figure 1:**
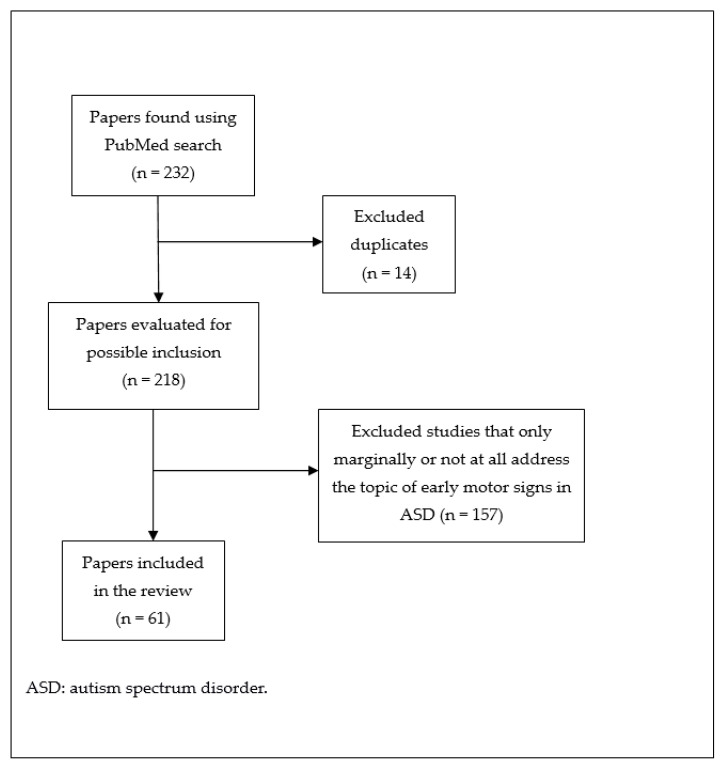
Paper selection flow chart.

A narrative review based on a search through PubMed (U.S. National Library of Medicine) was performed. The two following combinations of search terms joined by the Boolean operator “AND” were used: “infant” AND “motor” AND “autism spectrum disorder”; and “toddler” AND “motor” AND “autism spectrum disorder”. We considered all the papers found using these search terms published till September 2021, comprising reviews and original papers written anywhere in English. In this way, 212 and 20 papers were found respectively: after removing the duplicates, the total number of papers became 218. All these papers were read and those that concerned only marginally or not at all the topic of early motor function in ASD were excluded. Both authors of this review contributed to the selection of articles. Finally, 61 papers were included in this review (Figure 1). Additional relevant references, cited in the papers selected through PubMed or known by the authors, were considered. All papers included in the references of this review were read by both authors.

## 3. Results

Motor impairments are very important in ASD because they are possible early clinical markers and also because of their possible pathogenetic contribution to the development of social communication deficits in children with ASD, due to the basic role played by the motor system for exploring and knowing the surrounding environment [9]. Early motor dysfunction can manifest itself in ASD children as a mere delay in the acquisition on the motor domain or in the form of atypicalities of motor development. We will deal with both aspects. We will then discuss modern techniques to evaluate early motor dysfunction, the issue of children at high risk (HR) for ASD, the relationship between motor and social communication skills, and the effects of the treatment of motor dysfunction.

### 3.1. Early Motor Impairment in ASD Children: Delay of Motor Development

Already during the first year of life, a delay of both gross and/or fine motor abilities has been reported by several authors. As suggested by Arabameri and Sotoodeh, a delayed age of acquisition of sitting without support (mean months: 7.64), standing without support (mean months: 13.22), and walking alone (mean months: 18.31) has been found in ASD children [10]. Reindal et al. studied the age of first walking and its relationship with ASD symptom severity in a sample of 490 children (23% females), subdivided into ASD (*n* = 376) and non-ASD (*n* = 114) groups. ASD children achieved independent walking significantly later than non-ASD children (mean: 14.7 versus 13.8 months, respectively). Age of first walking turned out to be significantly associated with ASD symptom severity, and females showed a non-significant later age of first walking. The authors concluded that in children with delayed independent walking ASD should be considered for differential diagnosis, perhaps especially in females [11]. Bolton et al., performed a prospective longitudinal cohort study about the offspring of 14,541 women. According to the parents’ reports, children who later received a diagnosis of ASD already showed differences in fine motor skills, as well as in communication ones, from the age of 6 months [12]. Davidovitch et al. studied in the first 24 months of life the developmental trajectory of 335 low-risk (LR) infants who later received a diagnosis of ASD. They found that by 9 months of age ASD children started to fail the communication, as well as motor items compared to typical and delayed non-ASD children [13]. In the context of a longitudinal birth cohort study, Elberling et al., assessed infant mental health and development from birth to the age of 10 months. Mental health outcome was studied in 1585 individuals aged 5–7 years. Overall development problems and specific oral-motor development problems were found to be predictors of ASD [14]. Pusponegoro et al., performed a cross-sectional study considering gross motor and socialization skills in 40 ASD children aged 18 months-6 years and 40 age-matched typically developed (TD) controls. The mean gross motor score on the Vineland Adaptive Behavior Scales (2nd edition) was significantly lower in ASD children than in controls and the differences prevailed in skills requiring complex coordination such as ball throwing and catching, using stairs, jumping, and bicycling. Further, ASD children with gross motor deficits showed a mean socialization domain score significantly lower than those without gross motor impairments [15]. Oien et al., found that children who passed the M-CHAT (Modified Checklist for Autism in Toddlers) screening at 18 months and instead received later a diagnosis of ASD (so called false-negative cases) however presented delays and atypical features in social communication as well as fine motor domains at 18 months. Differences seemed to prevail in girls [16]. Among a general population sample of 515 infants (mean age 12.9 months), Kovaniemi et al., found that infants screened in the Of-Concern range on the ASD item cluster of the Brief Infant–Toddler Social and Emotional Assessment, administered to their parents, showed later achievement ages for gross motor skills than infants with the corresponding No Concern screen status [17]. Nishimura et al., found that ASD diagnosis may be predicted based on the neurodevelopmental trajectories during the first 2 years of life. The authors assessed neurodevelopment in 952 infants at seven time points up to the age of 24 months. At 32 months, ASD was diagnosed in 3.1% of the children. The authors identified five neurodevelopmental classes: high normal, normal, low normal, delayed, and markedly delayed. The probability of an ASD diagnosis was highest (32.6%) in the markedly delayed class in comparison with the others: respectively 6.4% and 4.0% for delayed and low normal classes, and 0% both in the normal and high normal classes [18]. Lebarton and Landa studied motor skills in 51 LR and 89 HR individuals aged 6 months [19]. Note that, in general, an infant is considered at HR for ASD if he/she has a sibling with ASD and/or was born preterm presenting low birth weight [8], however, in the literature, subjects at HR for ASD almost always mean individuals who have a family history of ASD in an older sibling. Among the 89 HR infants reported by Lebarton and Landa, 20 were later diagnosed with ASD. Results showed that motor development at age 6 months was correlated with ASD status at age 24–36 months, that is ASD was associated with lower early motor abilities [19]. Landa et al. studied prospectively and longitudinally social, language, and motor development in 235 HR and LR children, aged 6–36 months, subdivided into: ASD identified by 14 months (28), ASD identified after 14 months (26), and no ASD (181). ASD children exhibited a developmental level similar to non-ASD children at age 6 months, but thereafter they showed atypical trajectories. Impairment from 14 to 24 months prevailed in the early-ASD compared to the later-ASD group, but was similar at 36 months. About half of ASD children showed a period of around 2 years characterized by development of quantitative aspects within normal limits according to the results of standardized tests, but during this period development decelerated, typical social engagement decreased, and autistic symptoms emerged. During the ASD preclinical period the first signs of developmental disruption are likely to be nonspecific, involving, for example, communication or motor delay. The authors highlighted the importance of a developmental screening at regular intervals to be started by the time of the first birthday, in order to improve the early detection of the first signs possibly associated with preclinical ASD or with non-ASD delays. However, this study also highlights that the administration of standardized tests may not be sufficient to detect mild motor dysfunction [20]. Licari et al. evaluated through a prospective study the motor domain in 96 infants with early autism signs aged 9–14 months. At baseline motor difficulties were very frequent, affecting 63/96 infants (65.6%) in the gross motor domain and 29/96 infants (30.2%) in the fine motor domain. At a 6-month follow-up, 23/63 infants (36.5%) maintained gross motor difficulties, while 20/29 (69.0%) infants continued to show fine motor difficulties. Lower fine motor skills at baseline and follow-up were associated with greater severity of autism signs. The results underline the potential clinical value of motor skills’ evaluation within early autism screening [21]. Sasayama et al. studied 1067 children who had been screened for ASD at the age of 18 months. At age 6 years, 3.1% of them were diagnosed with ASD. Higher rates of difficulties in motor abilities as well as in social communication skills were found in ASD children at 18 months of age [22]. However, early motor dysfunction does not appear to be exclusive to ASD, as suggested by Hirota et al., who studied prospectively charted developmental milestones’ data obtained from home-based records in 720 children aged 5 years. All 720 children were evaluated to ascertain a possible diagnosis of neurodevelopmental disorder (NDD), including ASD, and 124 of them received a diagnosis of ASD, while 331 of them received a diagnosis of non-ASD NDD. Compared to children without NDD diagnosis, those with NDDs showed greater rates of potential delays in developmental domains, including also the motor domain, at as early as 12 months of life or even earlier. No significant differences were found between ASD and non-ASD NDD groups concerning the motor domain [23]. Language regression is reported in about 25% of ASD children. Manelis et al., in a sample of 218 ASD children, identified 36 cases who showed definite language regression and compared them to 104 cases without regression. The age at which ASD cases with language regression reach key developmental milestones such as crawling, walking, and first words was significantly younger than the age of cases without regression and similar to that of TD children. Yet, despite this, children with language regression were diagnosed with more severe ASD symptoms than children without regression [24]. The link between autism and motor delay is suggested not only by clinical data, but also by genetic data. Takahashi et al. studied in 734 children from the general population the possible association of the polygenic risk score for ASD (representing an estimate of the genetic liability to ASD) with neurodevelopmental progress. They found that genetic risks for ASD might be related to delays in the gross motor domain as well as in the receptive language domain (See Table 1 for a Summary) [25].

### 3.2. Early Motor Impairment in ASD Children: Atypicalities of Motor Function

For the purposes of an early diagnosis of ASD, not only a possible delay in the acquisition of motor skills should be taken into consideration, but also and perhaps above all the possible presence of atypical motor patterns, which are clearly more difficult to observe than a mere developmental delay during the clinical practice particularly with younger children. From 9 weeks gestational age to 21 weeks post-term, so called general movements (GMs) are a pattern of spontaneous movements performed without external stimulation, at first with the appearance of writhing movements (elliptical in form), then, from around 9th week post-term, with the appearance of fidgety movements (circular in form). Alterations of GMs are generally suggestive of an impairment of the central nervous system. Phagava et al. performed a retrospective study by analyzing the home videos of 20 infants later diagnosed as ASD. Compared to controls, ASD infants showed more often a poor repertoire of writhing GMs (with a lack of variable sequences, amplitude, and speed) as well as abnormal or absent fidgety movements [4]. Einspieler et al., reviewed literature, finding that 17 out of 25 ASD individuals (68%) and 100% of 17 individuals with Rett syndrome showed abnormal GMs during the first 5 months of life [26]. Zappella et al., through a retrospective study of home videos, recorded between birth and 6 months of life, compared the early development of eight males with transient autistic behaviors (lost after the age of 3) and that of ten males later diagnosed with ASD. Abnormal GMs were found significantly more frequently in infants later diagnosed with ASD than in those with only transient autistic behaviors. This was while eye contact, responsive smiling, and pre-speech vocalizations as well as concurrent motor repertoire including postures did not differentiate between the two groups [27]. Loh et al. studied longitudinally motor behaviors coded from videotapes at 12 and 18 months in eight siblings later diagnosed with ASD, in nine non-diagnosed siblings, and in 15 controls. They found that the ASD group ‘‘arm waved’’ more frequently at 12 and 18 months, while the ASD and non-diagnosed group showed one posture (‘‘hands to ears’’) more frequently than the controls at 18 months [28]. Morgan et al. analyzed the videotapes of 50 ASD infants aged 18–24 months compared to 25 infants with developmental delay and 50 TD ones. They found in ASD infants a higher rate and a larger inventory of repetitive and stereotyped movements both with objects (swiping, rubbing/squeezing, rolling/knocking over, rocking/flipping, etc.) and without objects (flapping, rubbing the body, etc.) [29]. Purpura et al., analyzing retrospectively home videos, found in ten ASD infants aged 6–12 months an increased frequency as well as duration of repetitive movements of upper and lower limbs bilaterally, compared to ten TD infants and ten with developmental delay. The authors suggested that particularly hands and fingers’ repetitive movements could be highly sensitive signs to consider in ASD early screening [30]. The ability of maintaining midline head position during early infancy has been considered. Gima et al., using video recordings, studied spontaneous movements at 9–20 weeks post-term age in 14 very low birth-weight infants who later developed ASD. They found that the percentage of midline head position was lower in the ASD group than in the TD group, suggesting that during early infancy poorer skill in maintaining midline head position may help to identify infants who later develop ASD [31]. Mitchell et al. carried out a review to identify the clinical markers for ASD in the first 2 years of life. They found, in addition to social communication deficits, also several atypical motor signs, which we mention in summary as follows, distinguishing them according to age. By 12 months of age, hypotonia and unusual posturing; atypical behaviors, such as hand flapping, finger flicking, shaking head and rolling eyes; delayed onset of independent sitting and walking; postural instability; head lag; impairment of fine motor skills. By 18 months of age: lower fine motor skills, perhaps also lower gross motor skills; reduced motor control; and postural instability. At 2 years, unusual postures, hypoactivity, and hypotonia; lower gross and/or fine motor skills; increased repetitive behaviors. This study highlights the heterogeneity of motor signs in these children, which at least partly depends on the age factor [32]. Body symmetry in infants can be involved, as pointed out by Esposito et al., who found that ASD infants may exhibit significantly reduced static and dynamic symmetry while lying in the first 5 months [33] or during unsupported gait when toddlers [34]. Sparaci et al. (2018) studied longitudinally 41 HR infants at 10, 12, 18 and 24 months of life. They assessed changes in grasp types and functional actions performed with a spoon during a tool use task in the context of a play-like scenario. Based on outcome and vocabulary evaluation at 36 months, infants were subdivided into: 11 HR with ASD, 15 HR with language delay, and 15 HR without delay. More HR without delay infants than HR infants with ASD performed grasp types facilitating spoon use at age 24 months and functional actions at age 10 months. In HR infants functional action production at 10 months predicted respectively word comprehension (12 months) and production (24 and 36 months). The results of this study suggest the presence of impairments in purposeful actions in infants going on to receive ASD diagnosis and of a relationship between functional action production and communication [35]. Also postures have been considered. Leezenbaum and Iverson studied prospectively early posture development in HR versus LR infants. They videotaped, respectively at 6, 8, 10, 12, and 14 months, 60 infants: 14 HR diagnosed with ASD (HR-ASD), 17 HR with language delay (HR-LD), 29 HR without diagnosis (HR-ND), and 25 LR. Compared to LR infants, HR-ASD ones and, to a lesser extent, HR-LD ones showed different postural trajectories characterized by slower development of more advanced postures. Further, subtle differences in posture sustainment were present between HR-ASD and HR-LD infants [36]. Serdarevic et al. studied longitudinally 2905 children in order to look for a possible association between infants’ neuromotor development and autistic traits in the general population. They examined overall motor development and muscle tone between ages 2–5 months. Parents rated their offspring autistic traits through two validated questionnaires. ASD diagnosis was confirmed in 30 children. The authors found that low muscle tone detected in infancy predicted autistic traits, while there was only a modest association between overall motor development and autistic traits [37].

Detection of motor signs can favor an early diagnosis of autism. Sacrey et al., in a prospective study, examined parents’ concerns about infants at HR for ASD at multiple time points during the first 2 years, finding that parents of HR children who were later diagnosed with ASD reported more concerns than parents of LR and HR children who did not receive ASD diagnosis. What interests us most about this study now is that concerns about sensory behavior and motor development predicted a subsequent ASD diagnosis as early as 6 months, while concerns about social communication skills and repetitive behaviors did not predict ASD diagnosis until after 12 months [38]. Matheis et al., in a sample of 1226 ASD children, found that the mean age of first concern of their parents was 13.97 months. The most frequent first concern was related to speech/language. We want to emphasize here that first concerns related to motor development predicted an earlier age of first concern, while, on the contrary, first concerns related to communication and speech/language predicted later age of first concern [39]. In line with what has just been reported are the results of Parmeggiani et al., who, in a retrospective cross-sectional study about ASD early features in 105 patients, found that motor skill disorders prevailed in children with age at onset in the first 12 months of life [40]. Chinello et al. studied, in a general population of infants aged 12–17 months, the relationship between the persistence of primitive reflexes involving hand and mouth use and infants’ motor repertoire, infants’ age, and subclinical autistic traits in their parents. They found that persistence of the primitive reflexes was related to a worse motor repertoire (including interaction with objects and people), irrespective of the infants’ age, and to subclinical autistic traits in their parents. The authors concluded suggesting the persistence of primitive reflexes as a marker for ASD early identification [41]. Further, according to Setoh et al., their findings about infants’ parents suggest that subclinical social communication anomalies may be related to lower motor performances in the next generation [42]. Harris’ review pointed out that early motor delays (first year of life) may predict a diagnosis of ASD, but also of other neurodevelopmental disorders including intellectual disability [43]. The possible diagnosis of ASD should be considered in infants showing motor delays or other concerning motor behaviors. In this regard she suggested screening for: fine motor delays at age 6–15 months and gross motor delays at age 3–10 months; the presence of motor stereotypies (such as hand flapping at age 18–24 months and atypical limb movements while walking at age ≤24 months); motor control abnormalities, such as head lag at age 6 months, delays in bringing hands to midline at age 4–6 months, delays in protective extension reactions while sitting, and in moving freely when sitting at around 6 months [43]. Sacrey et al. studied the motor act of reaching-to-grasp in children at HR and LR for ASD between 6 and 36 months of age. At 36 months, all children underwent a standardized diagnostic assessment, leading to a subdivision into three outcome groups: HR children with ASD diagnosis (10), HR children without ASD diagnosis (10), and LR children without ASD diagnosis (10). HR children with ASD showed higher, i.e., worse, total scores on the reach-to-grasp movement and higher scores on the components of orient, lift, and pronate compared to the other two groups [44]. Considering the motor assessment in ASD children, in their overview paper Whyatt and Craig outlined the progression made from initial, broad evaluation through clinical tools such as the Movement Assessment Battery for Children (M-ABC2) to following targeted kinematic assessment. According to the authors, kinematic results showed by literature underline impaired perception-action coupling to adapt movement to task demands, leading to rigid motor profiles. Motor dysfunction may be a core feature of ASD, related to a problem with temporal control caused by impaired perception-action coupling (See Table 2 for a Summary) [45].

### 3.3. Modern Evaluation of Early Motor Function

A clinical evaluation of motor function performed retrospectively or prospectively possibly using standardized tools, however careful and thorough, may not recognize subtle early motor signs. This has led to the development of techniques capable of providing quantitative measures of motor behavior and allowing possible more objective methods of assessment of subtle early motor signs. Some examples follow. Martin et al. conducted a quantitative study of head movement dynamics in 42 children aged 2.5–6.5 years, respectively, 21 with and 21 without ASD, through automated, computer-vision based head tracking. ASD children, compared to those without ASD, showed greater yaw displacement, i.e., greater head turning, and higher speed of yaw and roll, i.e., faster head turning and inclination. Note that head movement differences were specific to a social condition [46]. Dawson et al. using computer vision analysis, assessed midline head postural control in 104 toddlers (age: 16–31 months), 22 of whom received a diagnosis of ASD, while watching movies including social and nonsocial stimuli. ASD toddlers showed a higher rate of head movement when compared to non-ASD toddlers, indicating difficulties in maintaining head midline position while engaging attentional systems [47]. Caruso et al. studied the early motor trajectories of HR infants using the software MOVIDEA, developed to analyze videos providing objective kinematic features of infants’ movements. They used MOVIDEA applying it to video recordings of spontaneous movements of 50 HR and 53 LR infants collected respectively at 10 days and 6, 12, 18, and 24 weeks. Considering the clinical outcome, 18 HR infants received a diagnosis of NDD, whereas 32 HR and 53 LR infants were TD. The authors found that HR infants later diagnosed with NDD presented higher general motor activity associated with lower variability and velocity, as well as higher acceleration of global movement in the space. Furthermore, these infants showed patterns of higher periodicity of limbs, particularly the upper ones, during the first 12 weeks of life [48]. Wilson et al. utilized in a longitudinal study Opal wearable sensors to assess full day motor activity in five HR infants at 3, 6, 9, 12 months of life, thus obtaining a motion complexity measure. Motion complexity is critical to a typical motor development and its lack might indicate the presence of repetitive motor behaviors, which is a core ASD sign. The authors found that the two HR infants later diagnosed with ASD present lower motion complexity than the three that do not. This study provides interesting data, but evidently it is based on a very limited number of cases (See Table 3 for a Summary) [49].

### 3.4. Children at High Risk (HR) for ASD

Atypical motor development is often described also in infants at HR for ASD, even when (and this is the most likely occurrence) they are not later diagnosed with ASD. In a prospective/longitudinal study, Nickel et al. analyzed postural development of 22 HR infants, videotaped at age 6, 9, 12, and 14 months. These infants showed, compared to 18 age-matched LR infants, delay in achieving more advanced postures, moving freely within recently achieved postures (e.g., while sitting), and moving from one posture to another [50]. Achermann et al., through a three-dimensional motion capture technology, studied the way 10-month-old HR infants catch a ball rolling toward them, a task requiring adequate planning and execution. Several early motor measures were different in 39 HR infants in comparison with 19 controls. However, they were not related to autistic symptoms at 2 years, but to the following non-social, general development [51]. Other specific manual motor behaviors have been considered in HR individuals. Begum Ali et al. studied body midline crossing at age 5, 10, and 14 months in 81 infants with HR for ASD, 31 with HR for Attention Deficit Hyperactivity Disorder (ADHD: another neurodevelopmental disorder according to DSM-5 [1]), 20 with HR for both ASD and ADHD, and 29 with LR for either ASD or ADHD. They found that only at 10 months, individuals at HR for ASD and/or ADHD showed fewer manual midline crossing compared to LR infants; midline crossing was not related to ASD traits, but to ADHD traits at age 2 years. The authors hypothesized that these results may be due to disrupted multisensory integration abilities and attention shifting in the first year of life [52]. Leonard et al., examined the motor development of 20 children at HR for ASD, at age 9 and 40 months. All children underwent a series of motor, face processing, IQ (Intelligence Quotient) and diagnostic assessments at age 5–7 years during a follow-up visit, when only one subject had an ASD diagnosis. A greater proportion of subjects than expected showed motor problems at age 5–7 years and those reported by parents as having early poor motor skills were more likely to show lower face processing skills and higher social deficits at 5–7 years. The authors concluded that early motor problems may be a risk factor for later impairments of social communication and cognition [53]. Garrido et al., in their meta-analysis reviewed studies about linguistic and/or motor abilities in HR for ASD children compared to LR for ASD children. Ultimately, they considered 34 eligible studies that included 2376 children at age 12 months (64% HR versus 36% LR), 3764 at age 24 months (66% HR versus 34% LR), and 3422 at age 36 months (63% HR versus 37% LR). Compared to LR children, HR infants had worse linguistic and motor (fine and gross) abilities, even though they did not show a homogeneous pattern of altered skills. These differences were detectable at the age of 12 months and seemed to persist until the age of 3 years. Differences in language abilities were greater than those in motor skills, particularly in the first year [54]. Iverson et al., examined the gross and fine motor skills at 6 months in a large sample of 437 HR infants with heterogeneous developmental outcomes and in 188 infants with LR for ASD. Fine, but not gross, motor performance distinguished HR infants from LR infants. At 6 months fine, but not gross, motor performance predicted autism severity in the HR group at 36 months. These findings suggest the presence of early motor delays in HR infants, regardless of their developmental outcome. But relatively little is known about the nature of these delays. As pointed out by the authors, a limitation of this study (as well as of the others that have addressed these issues) is that a scale was used to assess the development of the infant which provides data on the presence or absence of motor behaviors, but not on which behaviors are present when an infant fails an item [55]. According to the review of Varcin and Jeste, prospective, longitudinal studies about infants at HR for ASD have showed that ASD behavioral signs are usually not detected until the second year of life, whereas developmental signs during the first year, including motor impairment, are often subtle and outside the ASD core signs [56]. The conclusions of these authors are almost entirely in line with those of Sacrey et al., according to which prospective studies about HR infants suggest that social communication deficits and repetitive behaviors appear during the second year of life, whereas additional features such as motor and sensory abnormalities appear already in the first year [57]. Taffoni et al. studied longitudinally the motor planning development in 19 HR for ASD and in 14 LR children through a shape sorter task at 14, 18, 24, and 36 months of life. According to behavioral and kinematic data, all performance improvements in this type of task depended on several critical developments, including increased motor and perceptual competence. There were no differences between HR and LR cases, but a descriptive analysis of data regarding three HR children later diagnosed with ASD suggested the presence of early onset differences in motor planning skills: see children’s action (such as reaching time and acceleration) and performance (such as the adjustment of the shapes) [58]. Landa et al., assuming that integration between visual input and motor output is crucial not only for improving motor abilities, but also for imitating and interpreting the actions of others, studied visual-motor coupling, or action anticipation, through the assessment of an interactive ball-rolling activity in 66 HR and 43 LR infants at age 6 months. Both LR and HR infants showed context appropriate looking behavior before and during the ball’s trajectory toward them, but, compared to LR infants, HR ones were less likely to show context appropriate anticipatory response to the approaching ball by moving their arm/hand to intercept it. Further, in the HR group there was an atypical predictive relationship between anticipatory response at age 6 months and predilection for looking at faces compared to objects at age 14 months. The authors concluded pointing out that the skills underlying anticipatory response are necessary for the development of internal action models, which likely are important to social development (see, for example, imitation as well as production of interpretable and well-timed interpersonal actions) [59].

Is there a counterpart to motor dysfunction at the level of brain networks? Marrus et al., using resting state fcMRI, studied the functional brain networks involved in walking and gross motor development in a mixed cross-sectional and longitudinal sample of 130 HR and LR infants. At age 12 months, functional connectivity of motor and default mode networks was involved in walking, whereas at age 24 months dorsal attention and posterior cingulo-opercular networks were implicated. Examination of general gross motor function also showed an involvement of motor and default mode networks at age 12 and 24 months, whereas dorsal attention, cingulo-opercular, frontoparietal, and subcortical networks were additionally involved at age 24 months (See Table 4 for a Summary) [60].

### 3.5. The Relationship between Motor and Social Communication Skills

From literature data growing evidence emerges that motor and communication (both verbal and non-verbal) skills are connected. Developmental changes in motor skills modify the way children interact with people and objects (e.g., by showing) and they may affect language development [61]. According to Leonard et al., the skill of exploring the environment, manipulating and sharing objects with others, stimulates the initiation of joint attention and modifies the types of parent’s vocalizations and expressions received by the infant [53]. Bradshow et al. studied the relationships between motor and social communication skills in 199 infants aged 12 months: 86 HR for ASD and 113 LR for ASD (TD). Infants were subdivided into: walkers, standers, or pre-walkers. HR walkers showed higher social communication skills, but similar cognitive skills, in comparison with HR pre-walkers. On the contrary, regardless of walking status, social communication and cognitive abilities were largely comparable for LR infants. Based on these results, the authors concluded that independent walking may foster the development of social communication skills in HR infants. Symbolic play, gestures, and language were all significantly better developed in HR walkers than in HR standers and/or pre-walkers, but it remains to be understood how the ability to walk can contribute to the development of these skills [62]. Bruyneel et al. studied the links between motor, joint attention and language skills in LR (31) and HR (32) children. In both groups, fine and gross motor skills at age 10 months affected language (both comprehension and expression) at age 36 months directly and indirectly through joint attention at age 14 months. Problems in motor and joint attention skills prevailed in HR children than in LR ones. Therefore, early motor skills’ assessment in HR children can be indicative of language problems later, particularly when also difficulties with joint attention occur [61]. In a meta-analysis West, collecting data from 1953 ASD infants aged 3–42 months, found that infant motor skills differed significantly in ASD compared to TD infants and this discrepancy augmented as age increased. Collecting data from 890 ASD infants aged 6–43 months, the author found that within ASD, motor skills and communication are related. West suggested that efforts to monitor HR for ASD infants may be boosted by including motor skills’ assessments. Motor deficits may be more easily detected compared to core ASD signs [63]. Manwaring et al. studied the possible connections among gesture, fine motor, and language skills in 110 ASD children and in a control group of 87 non-ASD children (with developmental delays or with TD), aged 12–48 months. The results of this study support the hypothesis of an underlying construct of gesture use including fine motor skills and predicting concurrent receptive and expressive language in ASD young children and in non-ASD controls, as well as later receptive language in ASD children. This further supports the importance of motor and nonverbal communication strategies in early language learning [64]. Choi et al., performed a prospective, longitudinal study about early developmental trajectories of fine motor skills in relation to expressive language outcomes considering 71 HR infants without ASD, 30 HR ones later diagnosed with ASD, and 69 LR ones without ASD. Fine motor abilities were assessed at age 6, 12, 18, and 24 months while expressive language outcomes were assessed at 36 months. HR infants later diagnosed with ASD exhibited significantly slower growth in fine motor skills from 6 to 24 months, compared to TD infants. Also, fine motor skills at age 6 months predicted expressive language at age 36 months. The authors concluded that poor fine motor skills may be addressed early in life to improve children’s language outcomes [65]. According to Iverson, HR infants vary widely in motor as well as communication development and this variation seems to produce cascading effects on development. Advances in motor skills support advances in communication (including language) development. Some HR infants seem to be indistinguishable from LR peers, while others show early but transient development delays; the most relevant delays are detectable in HR-ASD infants. However, it remains to be seen whether differences in early motor and communication development detected in HR-ASD infants are indicative of general delays or are specific to ASD [66]. According to Iverson, one of the main developmental tasks of infancy is represented by exploration. The acquisition of new and more complex gross and fine motor abilities allows infants to obtain more information about the social and physical worlds. But if these advances are slowed, this potential for exploration and learning opportunities decreases. Motor development delays may provide very important diagnostic information as well as excellent opportunities to develop intervention strategies addressing simultaneously motor and communication skills [66]. Assuming that in TD infants walk onset is associated with increased language growth, West et al. studied whether this association may be disrupted in HR infants, a population with important heterogeneity in motor and language development. They analyzed receptive and expressive language across the transition to walking in 91 HR infants aged 8–18 months subdivided into three groups (no diagnosis, language delay, and ASD) and in 25 LR infants aged 9–15 months. Only infants later diagnosed with ASD did not show increased language growth after walk onset. The authors concluded that walk onset may play a diverse role in language development in TD and in ASD infants. Probably, walking onset affords all infants greater autonomy in making experiences with social partners and objects but this autonomy may lead ASD infants to diverse experiences than their TD peers [67]. MacDonald et al. studied motor skills in 159 children, respectively, with ASD (*n* = 110), with pervasive developmental disorder not otherwise specified (*n* = 26), and non-ASD (*n* = 23) aged 14–33 months. They found that both fine and gross motor skills predicted autism severity: children with poorer motor skills had greater deficits of social communication skills [68]. Lemcke et al., collected prospectively data from 76,441 mothers’ interviews about development and behavior of their children at 6 and 18 months. By the end of follow-up, 720 individuals with ASD and 231 individuals with intellectual disability (ID) were identified. At age 6 months, only few predictors in the area of social communication and motor development were found to prevail in the ASD and ID groups, while at age 18 months social, language, and motor skills were definitely delayed for both groups. However, signs that can distinguish ASD from ID were unclear [69]. A result that at least apparently contradicts those previously reported comes from the study of Ben-Sasson and Gill who evaluated the development of 76 toddlers at 13 and 30 months, while their parents were given the First Year Inventory (FYI) (a standardized questionnaire for ASD screening) at 12 months. At 30 months, about 23.7% of the children received a clinical diagnosis such as, for example, ASD or developmental delay. The authors found that motor skill decrease was associated with language skill increase, while higher FYI sensory-regulatory risk was associated with gross motor skill decrease. The authors hypothesized that infants with developmental problems may divert energy from an area to a greater degree due to their developmental deficits [70]. Lebarton and Iverson studied progresses in locomotion related to progresses in communication development in HR infants who are not later diagnosed with ASD at 36 months. Infants were assessed monthly between 5 and 14 months of age. The authors found an increased presence of gross motor skill delay from 5 to 10 months. Further, they found positive relations between sitting and gesture and babble onset, as well as between prone development and gesture onset. Therefore, they demonstrated the presence of links between gross motor and communication skills also in HR infants without ASD diagnosis [71]. In the Lebarton and Landa’s aforementioned work considering 51 LR and 89 HR individuals, motor development at 6 months predicted expressive language at age 30 and 36 months [19]. Wu et al., compared the relationship of receptive and expressive language skills with motor functioning in 38 ASD toddlers aged 24 to 36 months and their age-matched TD peers. They found significant positive correlations between language skills and motor functioning in the ASD and TD individuals. The ASD toddlers with language delay showed worse multidimensional motor functioning than the ASD toddlers with typical language development and the TD individuals. Moreover, the lower motor functioning in ASD toddlers could predict the risks of expressive and receptive language delay. The authors concluded suggesting the importance of motor-based treatments targeting language skills in ASD young children [72]. Tanner and Dounavi conducted a systematic review about the earliest (before 18 months) ASD symptoms considering only prospective studies. Early fine and gross motor delays showed consistent correlations with expressive and receptive language development by 24 months of age, suggesting once again a cascading effect of early motor skills on language (See Table 5 for a Summary) [73].

### 3.6. Treatment of Motor Impairment in ASD

As suggested by Lebarton and Landa, early motor interventions may reduce the negative impact of motor problems on early social communication skills [19]. West believes the current interventions for infants and toddlers that focus primarily on increasing social communication skills may be enhanced by promoting and integrating motor behaviors [63]. In their review, Busti Ceccarelli et al. studied the effects of interventions concerning fundamental motor skills in ASD children. They found data suggesting potentially significant advances in the motor domain after these interventions. Unfortunately, only a subgroup of the considered studies examined the possible effects in the social communication domain after the advances in the motor skills, showing not univocal but promising results. The authors concluded suggesting the inclusion of motor skills training within the intervention programs for ASD children [74]. Even more recently, Elliott et al., pointed out that a fundamental motor ability intervention might produce improvements in ASD individuals not only at motor level, but even on social, listening, turn-taking, and transition skills [75]. And in fact, there are research data suggesting that early motor exploratory skills are associated with expressive vocabulary at age 1, 2, and 3.5 years, with cognitive skills in toddlerhood and childhood, and also with later academic skills [55]. For infants with clinically relevant early motor delays, intervention should focus on fundamental motor skills developing in the first year of life. Appropriate, parent-delivered interventions for motor skills may have positive effects also on other domains’ skills such as face processing [55]. According to Tanner and Dounavi, the role of early fine and gross motor abilities for expressive and receptive language development has been largely documented, therefore the authors underline the importance, already in the pre-diagnostic phase, of an intervention that follows an interdisciplinary approach, including also physical therapy, and that favors the development of motor skills [73]. Treatment of infants with early motor deficits may be very important because achievements in these areas can modify infants’ earliest experiences. But, as suggested by Leezenbaum and Iverson, instead of focusing on motor or social communication skills separately, it is probably more useful to broadly improve the infant’s ability for exploratory experiences, emphasizing the reciprocal influence between infant and caregiver. This theory is sustained by research data about Early Start Denver Model, which follows a holistic approach in the treatment of very young ASD children [36]. However, the literature data on the effectiveness of an intervention aimed at increasing motor skills in children with ASD are still very scarce today and therefore much more research is needed to better understand the effects of this type of intervention on the various domains of the development of these children (See Table 6 for a Summary).

## 4. Discussion

First, we would like to mention the limitations of our narrative review that are fundamentally related to the presence of a possible bias in the selection of papers included, due to a partially inevitable subjective evaluation by the authors. However, a narrative review is methodologically indicated when the purpose of authors is giving a broad perspective about a topic (like the one covered in this paper) which is not so focused that it can be treated in a systematic review [76].

An important element that emerges from the literature we reviewed is that the early detection of motor signs in ASD infants may contribute to making a timely autism diagnosis. For this purpose, attention should be paid within one year of life to possible (usually slight and not specific) motor signs, which not infrequently occur even before the appearance of social communication abnormalities in ASD infants. This is even more the case when dealing with a child who is predisposed to developing autism, that is a HR child. In fact, a large number of studies highlight the relevance of the developmental monitoring of HR infants, particularly those showing early motor delay who are the most likely to develop an ASD. Hence, the importance of including items dedicated to motor signs in the tests/questionnaires for early autism screening. In this regard, it would be important to know not only whether certain typical motor behaviors are present or absent, but also, if they are absent, which behaviors are observable [55].

It should also be emphasized that a mere early delay in motor development seems to be a fairly non-specific sign, as it can be found not only in infants who will then develop ASD but also in those who will develop other neurodevelopmental disorders or even in those who will make up for the gap with their peers and will later have a normal development. What appears most characteristic of ASD, on the other hand, seems to be the presence of some early atypicalities of motor development, such as a higher rate and a larger inventory of stereotyped movements both with and without objects [29]. However, it should be underlined that these atypicalities are often much more difficult to detect than a mere motor developmental delay during a normal clinical evaluation. In this sense, the administration of the currently used standardized tests may not be sufficient to detect a mild motor dysfunction [20]. Motor signs, and in particular the early ones, in individuals with ASD should therefore be more valued within the diagnostic criteria of DSM-5 [1], where so far they have been confined to the rank of associated symptoms of ASD. In our opinion, these signs might be included in ASD DSM-5 criteria within the “Restricted, repetitive patterns of behavior, interests, or activities”, near to the sensory abnormalities [8].

Early motor dysfunction could be not only a clinical marker that suggests a diagnosis, because, in the opinion of some authors, it may also play a pathogenetic role in ASD. How can this happen? Early vulnerabilities in motor skills may produce cascading effects on later outcomes, importantly, also in domains other than motor one. During the first year of life, TD infants achieve a range of new motor skills that improve considerably their interactions with people and objects creating more opportunities for exploration of the surrounding environment. These motor skills, allowing through manipulation and exploration the inclusion of objects into the interactions with others, may play also a role in the development of joint attention [55]. Therefore, the early detection of motor signs in HR individuals, even when social communication deficits are not evident, represents a relevant warning signal that leads one to suspect the imminent appearance of an ASD clinical picture [8]. In this perspective, it is probably not by chance that the physical activity seems to be effective not only on motor skills but also on social-communication skills and behavior (see reduction of maladaptive and stereotypical behaviors) in ASD children and adolescents, improving their quality of life [77,78]. Yet, many aspects about early motor dysfunction in ASD still need to be clarified. For example, if it is true that a delay in motor development can favor the appearance of social communication deficits, why for example in individuals with infantile cerebral palsy, in which there is by definition an early, persistent and often severe motor deficit impairing the exploration of the surrounding environment, does autism occur only in a minority of cases [79]? Similar considerations could be made also with regard to various neuromuscular pathologies that cause a delay in the acquisition of autonomous walking or even a failure to acquire it, without this leading to the development of an autistic-like behavior [80].

What could be behind the motor impairment found in children with autism? A clear answer to this question does not currently exist and we are still at the hypothesis level. However, at the origin of motor dysfunction in ASD infants there might be an early impairment of long-range brain connectivity (see the findings showed by brain functional magnetic resonance) causing failure in multisensory integration negatively affecting motor development, and not a defined focal lesion of the central nervous system. In fact, the neurological examination of these individuals almost never shows focal signs [8], as well as brain magnetic resonance imaging, which, when performed, in most cases does not shows focal lesions. An impairment of multisensory integration has also been directly or indirectly implicated in the development of various other clinical features of ASD [81].

## 5. Conclusions

Clinical experience and literature data suggest the presence of early heterogeneous motor dysfunctions in ASD patients that may even precede the onset of the core signs of autism. In the perspective of a timely diagnosis, the presence of early motor signs can be an important clue, especially in an individual considered at HR for autism. However, till now, motor signs have been considered only as associated clinical features of ASD, according to DSM-5 [1]. A pathogenetic role of early motor dysfunctions in the development of autism can be hypothesized. From this derives the importance of an early enabling intervention aimed at improving motor skills, which could also have favorable effects on other domains of development.

## Figures and Tables

**Table 1:** Motor delay and ASD.

Already during the first year of life, a delay of both gross and/or fine motor abilities has been reported in ASD children. In particular, a delayed age of acquisition of sitting without support, standing without support, and walking alone has been found. Motor delay may precede socio-communicative deficits.

ASD: autism spectrum disorder.

**Table 2:** Atypical motor patterns and ASD.

For the purposes of an early diagnosis of ASD, also the possible presence of atypical motor patterns should be taken into consideration. Alterations of general movements were found more frequently in infants later diagnosed with ASD. In ASD infants aged 18–24 months a higher rate and a larger inventory of repetitive and stereotyped movements both with objects and without objects have been found. ASD infants may show reduced static and dynamic body symmetry while lying in the first 5 months or during unsupported gait when toddlers. Low muscle tone detected in infancy may predict autistic traits.

ASD: autism spectrum disorder.

**Table 3:** Modern evaluation of early motor function in ASD.

A clinical evaluation of motor function performed using standardized tools, however careful and thorough, may not recognize subtle early motor signs. This has led to the development of techniques (in particular computer vision analysis) capable of providing quantitative measures of motor behavior and allowing possible more objective methods of assessment of subtle early motor signs in ASD children.

ASD: autism spectrum disorder.

**Table 4:** Children at HR for ASD.

Atypical motor development is often described also in infants at HR for ASD, even when they are not later diagnosed with ASD. Prospective studies about HR infants suggest that social communication deficits and repetitive behaviors appear during the second year of life, whereas additional features such as motor abnormalities appear already in the first year.

ASD: autism spectrum disorder; HR: high risk.

**Table 5:** Motor and social communication skills.

Motor and communication (both verbal and non-verbal) skills are connected. Advances in motor skills support advances in communication development. Developmental changes in motor skills modify the way children interact with people and objects (e.g., by showing) and they may affect language development. Efforts to monitor HR for ASD infants may be boosted by including motor skills’ assessments. Motor deficits may be more easily detected than core ASD signs. One of the main developmental tasks of infancy is represented by exploration. The acquisition of new and more complex gross and fine motor abilities allows infants to obtain more information about the social and physical worlds. If these advances are slowed, this potential for exploration and learning opportunities decreases.

ASD: autism spectrum disorder; HR: high risk.

**Table 6:** Treatment of motor impairment in ASD.

Early motor interventions may reduce the negative impact of motor problems on early social communication skills. Instead of focusing on motor or social communication skills separately, it is probably more useful to broadly improve the infant’s ability for exploratory experiences, emphasizing the reciprocal influence between infant and caregiver. This theory is sustained by research data about Early Start Denver Model, which follows a holistic approach in the treatment of very young ASD children.

ASD: autism spectrum disorder.

## Data Availability

Not applicable.
